# The role of oxidative stress in the crosstalk between leptin and mineralocorticoid receptor in the cardiac fibrosis associated with obesity

**DOI:** 10.1038/s41598-017-17103-9

**Published:** 2017-12-01

**Authors:** Josué Gutiérrez-Tenorio, Gema Marín-Royo, Ernesto Martínez-Martínez, Rubén Martín, María Miana, Natalia López-Andrés, Raquel Jurado-López, Isabel Gallardo, María Luaces, José Alberto San Román, María González-Amor, Mercedes Salaices, María Luisa Nieto, Victoria Cachofeiro

**Affiliations:** 1 Departamento de Fisiología, Facultad de Medicina, Universidad Complutense de Madrid and Instituto de Investigación Sanitaria Gregorio Marañón (IiSGM), Madrid, Spain; 2 Cardiovascular Translational Research, Navarrabiomed (Miguel Servet Foundation), Instituto de Investigación Sanitaria de Navarra (IdiSNA), Pamplona, Spain; 3 Instituto de Biología y Genética Molecular, CSIC-Universidad de Valladolid, Valladolid, Spain; 4 Facultad de Enfermería y Fisioterapia, Salus Infirmorum. Universidad Pontificia de Salamanca, Madrid, Spain; 5 Servicio de Cardiología, Instituto Cardiovascular, Hospital Clínico San Carlos, Madrid, Spain; 6 Instituto de Ciencias del Corazón (ICICOR), Hospital Clínico Universitario de Valladolid, Valladolid, Spain; 7 Departamento de Farmacología, Facultad de Medicina, Universidad Autónoma de Madrid and Instituto de Investigación Hospital Universitario La Paz (IdiPAZ), Madrid, Spain; 8 Ciber de Enfermedades Cardiovasculares (CIBERCV). Instituto de Salud Carlos III, Madrid, Spain

## Abstract

We have investigated whether mineralocorticoid receptor activation can participate in the profibrotic effects of leptin in cardiac myofibroblasts, as well as the potential mechanisms involved. The presence of eplerenone reduced the leptin-induced increase in protein levels of collagen I, transforming growth factor β, connective tissue growth factor and galectin-3 and the levels of both total and mitochondrial of superoxide anion (O_2_^.^^−^) in cardiac myofibroblasts. Likewise, the MEK/ERK inhibitor, PD98059, and the PI3/Akt inhibitor, LY294002, showed a similar pattern. Mitochondrial reactive oxygen species (ROS) scavenger (MitoTempo) attenuated the increase in body weight observed in rats fed a high fat diet (HFD). No differences were found in cardiac function or blood pressure among any group. However, the cardiac fibrosis and enhanced O_2_^.^-levels observed in HFD rats were attenuated by MitoTempo, which also prevented the increased circulating leptin and aldosterone levels in HFD fed animals. This study supports a role of mineralocorticoid receptor in the cardiac fibrosis induced by leptin in the context of obesity and highlights the role of the mitochondrial ROS in this process.

## Introduction

Extracellular matrix (ECM) accumulation is a common response of the heart to different types of damage, including obesity. The development of cardiovascular fibrosis has been repeatedly reported in obesity in experimental and clinical studies, which is frequently accompanied by co-morbidities such as hypertension and diabetes that can favour the development of cardiac fibrosis^1–5^. Growing evidence indicates that myocardial fibrosis is one of the pivotal contributors to heart muscle dysfunction in obesity^3,4,6^. The excessive ECM deposit due to a large number of myofibroblasts, the cell mainly responsible for fibrosis, can cause an aberrant remodelling that favours functional alterations, since a reduced relaxing capability of the heart can increase its filling pressure and contribute to diastolic dysfunction.

Multiple factors have been proposed as being responsible for the increased accumulation of collagen content in the myocardium in the context of obesity, with leptin being one of these factors^1,7,8^. This adipokine is locally produced in the heart in both the epicardial fat and in the myocardium and its production is up-regulated in obese rats^1^. In a previous study, we have shown that the cardiac levels of leptin are associated with levels of total collagen content, collagen I and transforming growth factor (TGF)-β in diet-induced obesity in rats. Leptin is also able to stimulate the synthesis of collagen I and the profibrotic mediators TGF-β, connective transforming growth factor (CTGF) and galectin-3 through the increase of oxidative stress and the activation of PI3K/Akt in cardiac myofibroblasts from adult rats^1,9^. Oxidative stress is characterized by the overproduction of reactive oxygen species (ROS) with the mitochondria being the main source^10^. Leptin seems to exert more actions at cardiac levels because elevated circulating leptin levels are associated with left ventricular hypertrophy in patients with uncomplicated obesity^11^.

Aldosterone through binding of mineralocorticoid receptor (MR), triggers the development of cardiac fibrosis in different pathologies, and its pharmacological blockade has demonstrated reduced interstitial fibrosis in these situations^12–14^. Different studies have demonstrated that aldosterone is inappropriately elevated in obesity, and MR antagonism improves left ventricle function and reduces circulating procollagen levels in patients with obesity without other comorbidities^4,15^. Similarly, low doses of spironolactone showed an anti-fibrotic effect in obese rats^16^.

Interactions among leptin and aldosterone have been previously reported in different scenarios and at different levels. Leptin raises blood pressure and induces endothelial dysfunction via aldosterone-dependent mechanisms in obese female mice^17^. Regarding the fibrotic actions, it has been shown that leptin promotes cardiac fibrosis via MR-dependent mechanisms in control and leptin-deficient mice^5^. These data support a link between leptin and MR, which could result in the potentiation of the myocardial fibrosis associated with obesity. However, how or at which level these interactions occur is unknown. Therefore, the aim of this study was to investigate whether MR activation can mediate the profibrotic effects of leptin in adult cardiac myofibroblasts, the main cells involved in cardiac fibrosis^18^. In addition, we have explored the potential mechanisms involved in this process. For this purpose, we have performed *in vitro* and *in vivo* studies in adult cardiac myofibroblasts and in rats fed a high fat diet (HFD).

## Methods

Detailed methods are available in the online-only Data Supplement.

### Cell culture conditions

Cardiac fibroblasts were isolated from the heart of adult male Wistar rats and used between passages 4 and 5. All assays in the present study were done at a temperature of 37 °C, 95% sterile air and 5% CO_2_ in a saturation humidified incubator. Cells were treated with leptin (100 ng/mL, BioVendor, Germany) for 24 h in the presence or absence of the MR antagonist (eplerenone 10^−6^ mol/L; Sigma; St Louis, MO, USA), and in the presence or absence of the inhibitors of either PI3K or MEK pathways, LY294002 (20 × 10^−6^ mol/L) and PD98059 (25 × 10^−6^ mol/L), respectively.

### Animals

Male Wistar rats of 150 g (Harlan Ibérica, Barcelona, Spain) were fed either a high-fat diet (HFD, 35% fat; Harlan Teklad #TD.03307, Haslett, MI, USA; n = 16) or a standard diet (3.5% fat; Harlan Teklad #TD.2014; Haslett, MI, USA; = 16) for 6 weeks. Half of the animals of each group received either the mitochondrial antioxidant MitoTempo (0.7 mg Kg^−1^ day^−1^ Sigma, Louis, MO, USA) i.p. or vehicle (saline) from the third week on. The dose used of MitoTempo was chosen from previous publication^19^. Animal weight was controlled every week. Food and water intake were administered at libitum and determined throughout the experimental period. Blood and heart were collected at the end of the experiment. The Animal Care and Use Committee of Universidad Complutense de Madrid and Dirección General de Medio Ambiente, Comunidad de Madrid (PROEX 242/15) approved all experimental procedures according to the Spanish Policy for Animal Protection RD53/2013, which meets the European Union Directive 2010/63/UE.

### Statistical analysis

Data are expressed as mean ± SEM. Normality of distributions was verified by means of the Kolmogorov–Smirnov test. Pearson correlation analysis was used to examine association among different variables. Data were analyzed using a one-way analysis of variance, followed by a Newman–Keuls to assess specific differences among groups or conditions or unpaired Student’s t-test as corresponding using GraphPad Software Inc. (San Diego, CA, USA). The predetermined significance level was p < 0.05.

## Results

### Mineralocorticoid receptor blockade reduces the ECM production and oxidative stress induced by leptin

**Figure 1:**
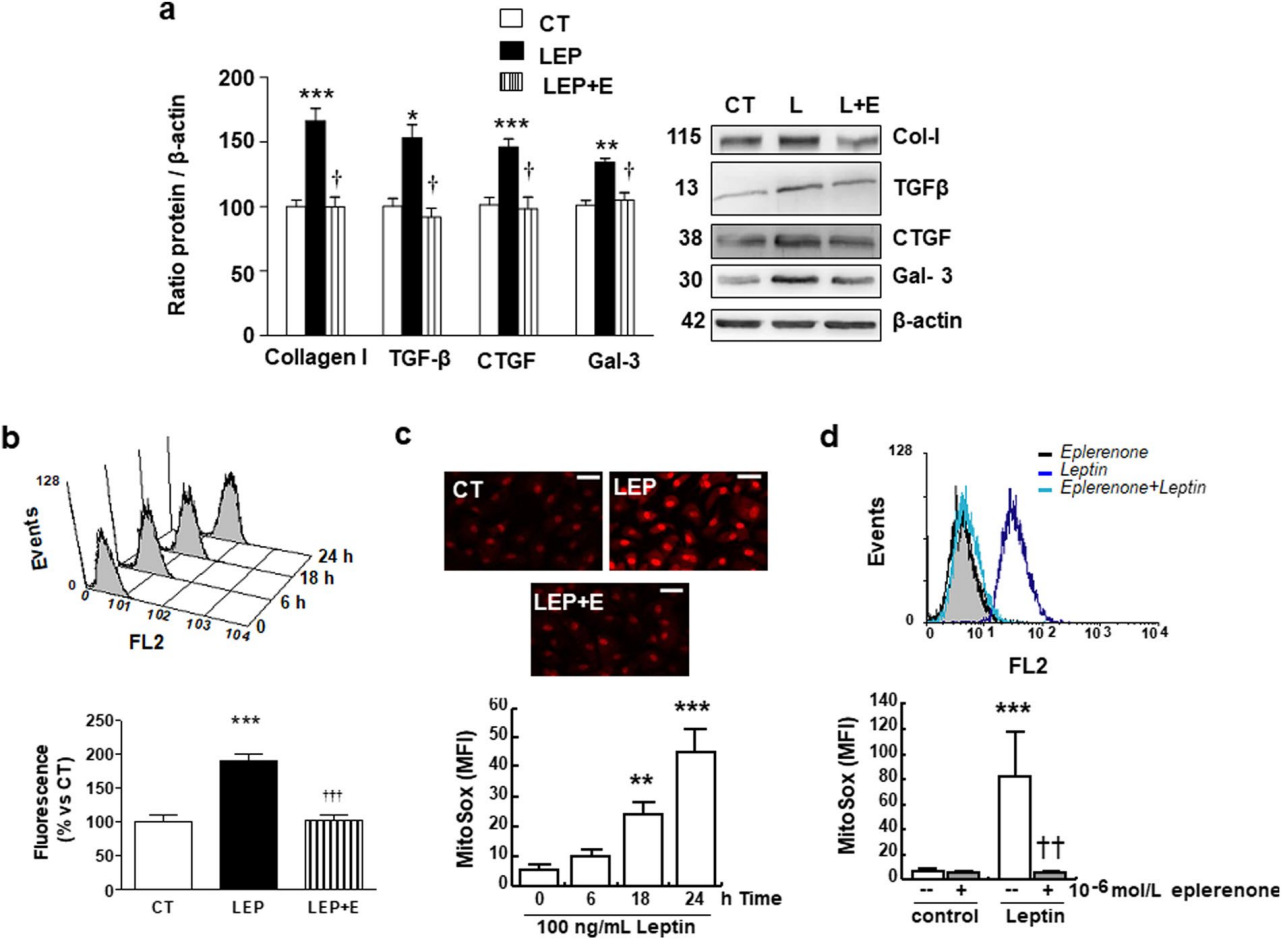
Impact of the mineralocorticoid receptor antagonist eplerenone on profibrotic protein factors and ROS levels in cardiac myofibroblasts. Cardiac myofibroblasts stimulated for 24 hours with leptin (100 ng/mL) in the presence (LEP) or absence (CT) of the mineralocorticoid receptor antagonist (eplerenone; 10^−6^ mol/L; L + E) were analyzed. (**a**) Protein levels of collagen type I, TGF-β, CTGF and galectin-3. (**b**) Time course of mitochondrial ROS generation in leptin-treated cells labeled with Mitosox: Representative histogram and quantification. (**c**) Representative microphotographs in cells labeled with DHE analyzed by fluorescence microscopy and quantification of total superoxide anions production induced by leptin in presence or absence of the mineralocorticoid receptor antagonist eplerenone (10^−6^ mol/L) (magnification 40X). (**d**) Mitochondrial ROS production in presence or absence of the mineralocorticoid receptor antagonist eplerenone (10^−6^ mol/L): Representative histogram and quantification. Untreated cells (solid black curves) were compared with cells treated with leptin (solid dark grey curves) or with eplerenone + leptin (open grey curves) for 24 h. Scale bar 50 µm. Bar graphs represent the mean ± SEM of 4 assays, in arbitrary units normalized to β-actin. *p < 0.05; **p < 0.01; ***p < 0.001 *vs*. control (CT). ^†^p < 0.05; ^††^p < 0.01; ^†††^p < 0.001 *vs*. leptin (LEP). Uncropped images of the blots for Fig. 2a are shown in supplementary Fig. 6.

As shown in Fig. 1a, eplerenone was able to reduce the collagen I production induced by leptin in cardiac myofibroblasts. Similarly, eplerenone was able to prevent the production of the profibrotic mediators involved in the collagen production induced by this adipokine: TGF-β, CTGF and galectin-3 (Fig. 1a).

Leptin increased mitochondrial superoxide anion (O_2_^.^^−^) production in both a dose- (data not shown) and a time-dependent manner (Fig.1b) as well as total O_2_^.^^−^ (Fig. 1c) in cardiac myofibroblast. Eplerenone reduced oxidative stress in cardiac myofibroblast by reducing total (Fig.1c) and mitochondrial O_2_^−^ levels (Fig.1d). Eplerenone alone was unable to affect any of the studied parameters (data not shown).

Likewise, leptin induced nitrosative stress in cardiac myofibroblasts, which was also diminished by the presence of eplerenone. As shown in Fig. S1a and b, cardiac myofibroblasts treated with leptin had detectable levels of intracellular nitric oxide (NO), which increased in a time-dependent manner. Untreated cells were mostly negative for the presence of NO. The presence of eplerenone abolished leptin-evoked NO production (Fig. S1c)

We also investigated whether leptin stimulation affected mitochondrial membrane potential. Leptin treatment for up to 24 h did not induce a substantial decline in Rd123 fluorescence nor in JC-1 fluorescence, indicating that mitochondrial membrane potential was unaffected. In contrast, and as expected, H_2_O_2_ exposure triggered a dramatic decrease in Rd123 and JC-1 staining (Fig. S2).

### Involvement of Akt and ERK pathways in profibrotic and prooxidant effects of leptin

**Figure 2:**
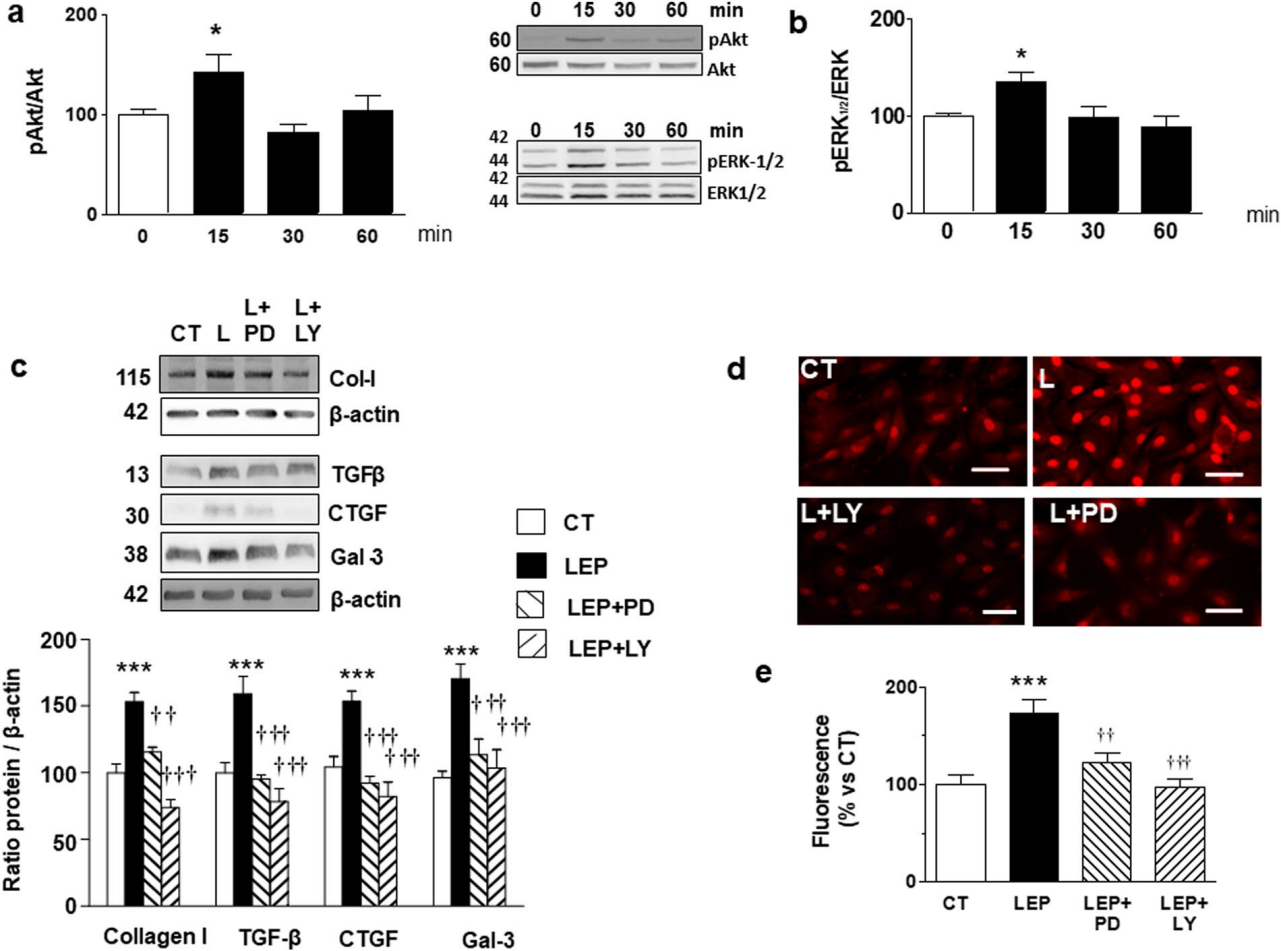
Effect of leptin on Akt and MEK pathways and impact of inhibition of Akt and MEK pathways on profibrotic protein factors and ROS levels in cardiac myofibroblasts. Protein levels of (**a**) pAkt/Akt and (**b**) pERK 1/2/ERK1/2 stimulated by leptin (100 ng/mL) for indicated time intervals. Cardiac myofibroblasts stimulated for 24 hours with leptin (100 ng/mL) in the presence or absence of the inhibitors of either MEK (PD98059; PD; 25 × 10^−6^ mol/L) or Akt (LY294002; LY; 20 × 10^−6^ mol/L) pathways for 24 hours. (**c**) Protein levels of collagen type I, TGF-β, CTGF and galectin-3. (**d**) Representative microphotographs in cells labeled with DHE and (**e**) Quantification of total superoxide anions and. Bar graphs represent the mean ± SD of 3–4 assays in arbitrary units normalized to β-actin. *p < 0.05; ***p < 0.001 *vs*. control. ^††^p < 0.01; ^†††^p < 0.001 *vs*. leptin.

Leptin was able to stimulate the phosphorylation of both Akt and ERK1/2 in cardiac myofibroblasts, reaching the maximum level at 15 minutes (Fig. 2a and b). This activation shows a biphasic pattern because it was also observed 24 hours after exposure to leptin (Fig. S3). Taking into consideration that PI3K/Akt and MAPK/ERK pathways could mediate the phosphorylation of the signal transducer and activator of transcription 3(STAT3) which regulates genes involved in ECM^20^, we evaluated this possibility. As shown in Fig. S4a, leptin was able to stimulate the phosphorylation of STAT3 in cardiac myofibroblasts, reaching the maximum level at 30 minutes (Fig. S4a). STAT3 phosphorylation was also stimulated by leptin at 24 hours and this effect was blocked by the presence of both pathway inhibitors (LY294002 and PD98059; Fig. S4B).

Next, we determined the role of these signaling mediators in profibrotic and prooxidant effects of leptin in cardiac myofibroblasts measured in cells pretreated with the selective inhibitors of these pathways PD98059 and LY294002, respectively. Both inhibitors reduced the production of collagen type I induced by leptin (Fig. 2c), as well as that of the mediators involved in this production TGF-β, CTGF and galectin-3 (Fig. 2c). Moreover, the presence of PD98059 and LY294002 was able to reduce the production of total ROS (Fig. 2d and e).

### Effect of leptin in activation of EGFR

**Figure 3:**
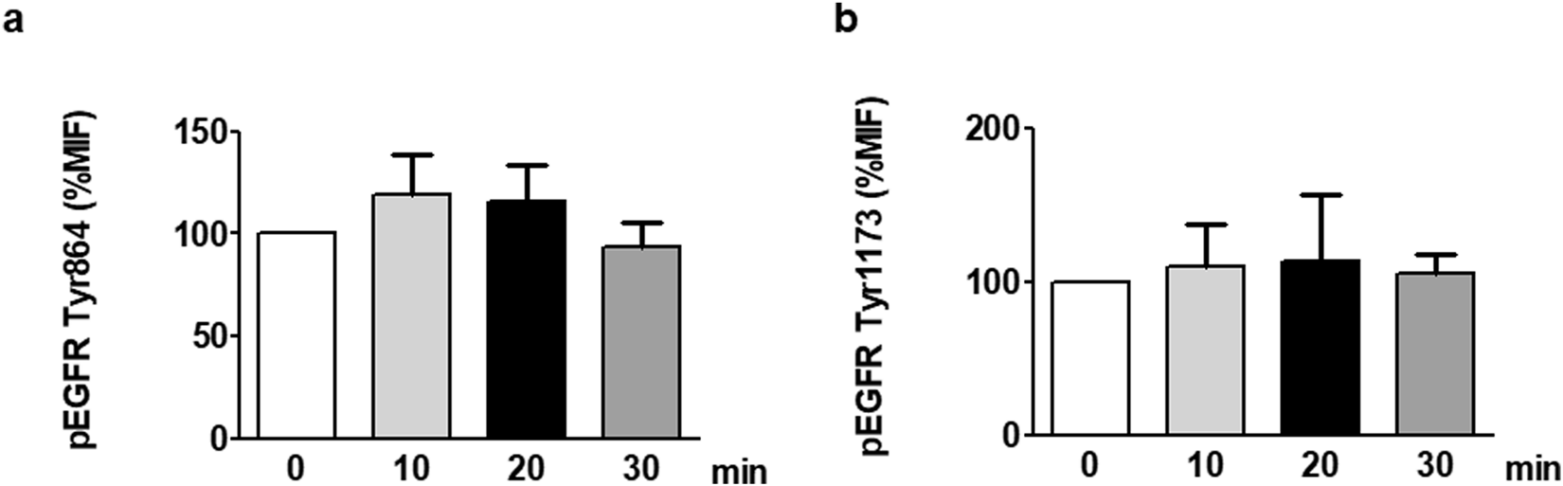
Effect of leptin on EGFR transactivation in cardiac myofibroblasts. EGFR phosphorylation in either Tyr864 (**a**) or Tyr 1173 (**b**) were analyzed by flow cytometry analysis in cardiac myofibroblasts stimulated with leptin (100 ng/mL) for indicated time intervals. Bar graphs represent the mean ± SEM of 3 assays.

In order to determine if the activation of the receptor EGFR is involved in the effects of leptin in cardiac myofibroblasts, we examined the EGFR phosphorylation status at Tyr845 and Tyr1176 in response to 100 ng/ml of leptin at different times. Data of Fig. 3a and b show that leptin does not produce a significant effect in EGFR activation.

### Interactions between mineralocorticoid receptor and leptin in the proliferation of cardiac myofibroblasts

In order to evaluate whether this interaction between MR and leptin not only involved ECM production but other actions, we explored if leptin is able to stimulate the proliferation of cardiac myofibroblasts in the presence or absence of aldosterone. Leptin alone or in the presence of aldosterone was unable to stimulate the proliferation of these cells (Fig. S5).

### Mitochondrial ROS have a role in cardiac fibrosis and leptin and aldosterone levels in diet-induced obese rats

**Figure 4:**
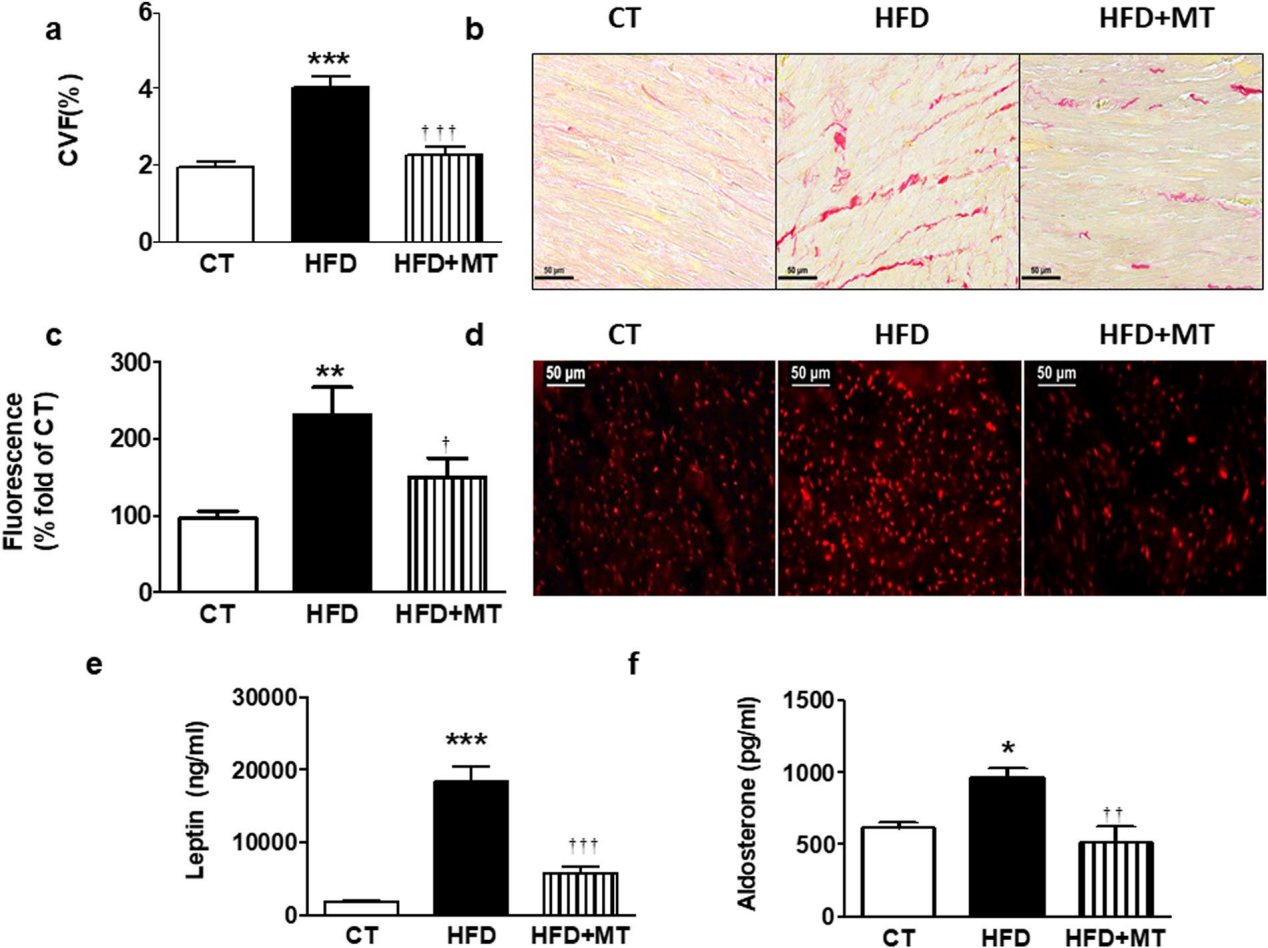
Impact of a mitochondrial ROS scavenger on cardiac fibrosis and ROS levels and plasma leptin and aldosterone levels in control and obese rats. Heart from rats fed a standard diet (CT) or a high fat diet (HFD) treated with the mitochondrial ROS scavenger (MitoTempo; MT; 0.7 mg Kg^−1^ day^−1^) were analyzed. (**a**) Quantification of collagen volume fraction (CVF). (**b**) Representative microphotographs of myocardial sections staining with picrosirius red examined by light microscopy (magnification 40X). (**c**) Quantification of superoxide anions production and (**d**) representative microphotographs of myocardial sections labeled with DHE analyzed by fluorescence microscopy (magnification 40X). Plasma levels of leptin (**e**) and aldosterone (**f**) of the same animals. Bar graphs represent the mean ± SEM of 6–8 animals. Scale bar: 50 µm. *p < 0.05; **p < 0.01; ***p < 0.001 *vs*. control group. ^†^p < 0.05; ^††^p < 0.01; ^†††^p < 0.001 *vs*. HFD group.

Taking into consideration that oxidative stress participates in the fibrotic effect induced by leptin and eplerenone reduced the oxidative stress induced by leptin, we evaluated whether oxidative stress is also involved in the possible interaction between MR and leptin observed *in vivo*. We also explored the effect of the administration of the mitochondrial ROS scavenger MitoTempo in rats fed an HFD. Body weight gain was expectedly significantly higher in rats fed an HFD as compared with rats fed a standard diet. This increase was smaller in animals treated with MitoTempo (Table 1). Similarly, MitoTempo reduced the increase in the body weight in obese animals (Table 1). It should be noted that the changes in body weight triggered by MitoTempo in HFD-fed rats were not consequence of a reduction in food intake (data not shown). Neither diet nor MitoTempo were able to modify systolic or diastolic function (Table 1). In addition, no differences in blood pressure levels were observed among any group along the study. Cardiac interstitial fibrosis was higher in obese animals than in controls (Fig. 4a and b). These levels were correlated with those of leptin (r = 0.8003, p < 0.001) and aldosterone (r = 0.6630, p < 0.01). The increase in cardiac fibrosis observed in HFD was prevented by the administration of MitoTempo. This antioxidant also prevented the altered cardiac O_2_^.^^−^ production in heart observed in HFD animals (Fig. 4c and d). In fact, cardiac ROS and fibrosis levels correlated with each other (r = 0.6634, p < 0.01).

**Table 1:** Effect of the mitochondrial reactive oxygen species scavenger (MitoTempo; MT; 0.7 mg Kg^−1^ day^−1^) on body weight, relative heart weight, echocardiographic parameters and systolic blood pressure in rats fed a standard diet (CT) or a high fat diet (HFD).

	CT	HFD	HFD + MT
Body Weight (g).	356.6 ± 10.7	442.8 ± 8.4***	397.8 ± 9.4 **^††^
HW/TL (mg/cm tibia)	24.7 ± 0.56	28.3 ± 1*	25.9 ± 0.7
IVT (mm)	1.51 ± 0.12	1.38 ± 0.5	1.4 ± 0.06
PWT(mm)	1.46 ± 0.1	1.37 ± 0.06	1.45 ± 0.06
EDD (mm)	6.88 ± 0.43	7.21 ± 0.23	6.81 ± 0.11
ESD (mm)	3.40 ± 0.36	3.98 ± 0.27	3.75 ± 0.3
EF (%)	86.9 ± 2.8	79.6 ± 3.1	78.8 ± 3.6
FS (%)	48.5 ± 3.2	42.3 ± 3.3	44.4 ± 3.8
E/A ratio	1.96 ± 0.17	2.03 ± 0.17	1.91 ± 0.12
SBP (mmHg)	133.7 ± 2.5	138.1 ± 2.1	133.9 ± 2.6

HW: heart weight; TL: tibia length; IVT: interventricular septum thickness; PWT: posterior wall thickness; EDD: end-diastolic diameter; ESD: end-systolic diameter; EF: ejection fraction; FS: fractional shortening; E/A ratio; SBP: systolic blood pressure. Data values represent mean ± S.E.M of 8 animals. *p < 0.05; **p < 0.01; ***p < 0.001 *vs*. control group. ^††^p < 0.01 *vs*. HFD group.

Leptin levels were expectedly higher in obese animals than in controls, which were reduced in those animals treated with MitoTempo (Fig. 4e). Aldosterone plasma levels show a similar pattern to those observed with leptin (Fig. 4f). In fact, a correlation was observed between both leptin and aldosterone levels (r = 0.6229, p < 0.01). In addition, both leptin (r = 0.6237; p < 0.05) and aldosterone levels (r = 0.7861, p < 0.001) were correlated with those of cardiac ROS. The administration of MitoTempo did not modify any of the evaluated parameters in control animals (data not shown).

## Discussion

The purpose of this study was to investigate the involvement of MR in the ECM production induced by leptin in cardiac myofibroblasts, which participates in the cardiac fibrosis associated with obesity^1^. We herein report that the MR antagonist eplerenone prevents the increase in collagen I synthesis induced by this adipokine. Eplerenone also reduced oxidative stress, TGF-β, CTGF and galectin-3 levels which are involved in the ECM production induced by leptin^1,7,8^. These results might suggest that the cardiac fibrotic effect of leptin could involve MR activation through oxidative stress-dependent pathway which activates the down-stream mediators by the activation of PI3K/Akt and MAPK/ERK pathways. Likewise, the *in vivo* data also show that the administration of a mitochondrial ROS scavenger in rats fed an HFD reduced cardiac fibrosis, as well as leptin and aldosterone plasma levels, supporting that there is an association among fibrosis, oxidative stress, leptin, and MR in obese normotensive rats. These data could help to understand the complex scenario involved in the development of cardiac fibrosis in the context of obesity.

The present data suggests an interaction between leptin and MR, which seems to be relevant in the development of cardiac fibrosis in the context of obesity in which both play a significant role^1,4,5,7,21,22^. This affirmation is based on two facts: First, the presence in the incubation media of eplerenone reduced the increase in collagen type I induced by leptin in cardiac myofibroblasts, the main cells involved in cardiac fibrosis^18^. Second, eplerenone prevented the production of the end effectors of leptin-induced ECM production (TGF-β, CTGF or galectin-3) in these cells^1^. In addition, a correlation between aldosterone levels and total cardiac collagen was observed in normotensive animals fed an HFD in the presence of high leptin levels. Moreover, the reduction of cardiac fibrosis induced by the mitochondrial ROS scavenger MitoTempo in animals fed an HFD was accompanied by a reduction in both leptin and aldosterone plasma levels. We have previously reported the role of oxidative stress in the fibrotic effect induced by leptin in cardiac and vascular fibrosis^1,23^. Therefore, the data suggest that leptin through ROS production can facilitate MR activation, which can mediate ECM production induced by this adipokine. In fact, this interaction seems to be more complex because eplerenone was able to reduce oxidative stress induced by leptin independently of its origin or nature (total ROS, nitro-oxidative species or mitochondrial ROS) in cardiac myofibroblasts, supporting the notion that there exists a vicious circle between oxidative stress and MR.

It is necessary to mention that we cannot exclude the possibility that the reduction in body weight gain, which was not consequence of a reduction in food intake, elicited by MitoTempo in HFD animals could also be partially responsible for the reduction in cardiac fibrosis and in leptin and aldosterone levels observed in the treated group. A similar finding has been reported with another mitochondrial ROS scavenger (MitoQ) in obese rats^24,25^. However, MitoQ was able to reduce cardiac fibrosis in a model of cardiac toxicity in rats without body weight change^26^ or reduced Akt activation in rat cardiac myocyte^27^. Total antioxidant capacity in the diet has been found to be inversely related to central adiposity, metabolic and oxidative stress bio-markers, and risk for cardiovascular diseases^28^. Therefore, it could be proposed that the observed effect on body weight could also be a consequence of the antioxidant capacity of MitoTempo. Given that changes in food intake were not observed further investigation focusing on lipid absorption, metabolism and/or accumulation are required to understand the underlying mechanisms involved in the in the sliming effect.

Consistent with previous studies in different conditions^23,29–34^, leptin stimulates PI3K/Akt, STAT-3 and MAPK/ERK signaling mediators in cardiac myofibroblasts that seem to be involved in the ECM production in cardiac myofibroblasts caused by this adipokine. This affirmation is based on the fact that the presence of their inhibitors blocked the synthesis of collagen I and the downstream–mediators (TGF-β, CTGF and galectin-3) induced by leptin. Therefore, taking into consideration that phosphorylation of PI3K/Akt and MAPK/ERK is involved in the fibrotic effects of both leptin and mineralocorticoid receptor activation^32,34–37^, these data suggest that PI3K/Akt and MAPK/ERK can act as common signaling mediators for both factors, regulating downstream events which lead to the production of end effectors, including TGF-β, CTGF and galectin-3, and which finally lead to the synthesis of ECM in cardiac myofibroblasts. The possible interaction between leptin and MR signaling pathways leading to PI3K/Akt and MAPK/ERK activation may occur at several different stages and their understanding warrants a more detailed analysis

The EGFR family and its ligands serve as a switchboard for the regulation of multiple cellular processes, including fibrosis. EGFR transactivation in cardiac cells has been observed following stimulation with leptin after MR activation, and its action has been implicated in cardiac remodelling^38–41^. EGFR transactivation is also involved in ROS generation, induced by high concentrations of glucose in rat cardiomyocytes^42^ and supporting that transactivation of EGFR plays a central role in mediating cardiac damage. However, our data does not support the phosphorylation of EGFR as a signaling which engages in leptin-mineralocorticoid receptor crosstalk in ECM production in adult cardiac myofibroblasts.

In summary, our data show that the cardiac fibrotic effect of leptin could involve the MR activation through oxidative stress-dependent pathway which activates the PI3K/Akt and MAPK/ERK pathways and consequently the production of end effectors, including TGF-β, CTGF and galectin-3, which are mainly responsible for the final synthesis of ECM in cardiac fibroblasts. These findings will all together aid in understanding the crosstalk among these factors in the production of ECM, which plays a role in the development of cardiac remodelling in the context of obesity and support the relevance of mitochondrial oxidative stress in this process. Therefore, the study highlights the complexity of the molecular mechanisms involved in the development of myocardial fibrosis associated with obesity.

## Electronic supplementary material

Supplementary data
