# Supplementary material for: The role of oxidative stress in the crosstalk between leptin and mineralocorticoid receptor in the cardiac fibrosis associated with obesity
Source: Sci Rep. 2017 Dec 1;7:16802. doi: 10.1038/s41598-017-17103-9 (PMC5711898; doi:10.1038/s41598-017-17103-9)
Supplement: Supplementary file 1 — Supplementary data [file 41598_2017_17103_MOESM1_ESM.doc]

**The role of oxidative stress in the crosstalk between leptin and mineralocorticoid receptor in the cardiac fibrosis associated with obesity**

Josué Gutiérrez-Tenorio^1^*, Gema Marín-Royo ^1^*, Ernesto Martínez-Martínez^1,2^, Rubén Martín^3^, María Miana^1,4^, Natalia López-Andrés^2^, Raquel Jurado-López^1^, Isabel Gallardo^3^, María Luaces^5^, José Alberto San Román^6,8^, María González-Amor^7^, Mercedes Salaices^7,8^, María Luisa Nieto^3,8†^, Victoria Cachofeiro^1,7†^

^1^Departamento de Fisiología, Facultad de Medicina, Universidad Complutense de Madrid and Instituto de Investigación Sanitaria Gregorio Marañón (IiSGM), Madrid, Spain.

^2^Cardiovascular Translational Research, Navarrabiomed (Miguel Servet Foundation), Instituto de Investigación Sanitaria de Navarra (IdiSNA), Pamplona, Spain.

^3^ Instituto de Biología y Genética Molecular, CSIC-Universidad de Valladolid, Spain.

^4^ Facultad de Enfermería y Fisioterapia, Salus Infirmorum. Universidad Pontificia de Salamanca, Madrid, Spain.

^5^Servicio de Cardiología, Instituto Cardiovascular, Hospital Clínico San Carlos, Madrid, Spain.

^6^Instituto de Ciencias del Corazón (ICICOR), Hospital Clínico Universitario de Valladolid, Valladolid, Spain

^7^Departamento de Farmacología, Facultad de Medicina, Universidad Autónoma de Madrid and Instituto de Investigación Hospital Universitario La Paz (IdiPAZ), Spain.

^8^Ciber de Enfermedades Cardiovasculares (CIBERCV). Instituto de Salud Carlos III. Madrid. Spain

* and †: These authors contributed equally to this work.

**Methods**

*Cell isolation and cell culture conditions*

Cardiac fibroblasts were isolated from the heart of adult male Wistar rats (weighing 150 g; Harlan Ibérica, Barcelona, Spain) by differential centrifugation of cardiac cells after enzymatic digestion of the hearts utilizing a mixture of collagenase-trypsin. As previously reported, the characterization of the cells using immunocytochemistry revealed expression of vimentin and α-smooth muscle cell actin.^1^ A consistent coexpression of both antigens was observed through passage 1, indicating that the cells possessed a myofibroblast phenotype. Cells were used between passages 4 and 5. The cells were maintained in DMEM medium supplemented with 10% FBS, 10x10^-3^ mol/L L-glutamine, 100 U/mL penicillin/streptomycin, 2 x10^-3^ mol/L L-pyruvate and 10 x10^-3^ mol/ L HEPES. The cells were seeded at a density of 0.5 × 10^6^ cells in a T- 175 tissue culture flask and then grown as monolayer culture. Cells were passaged with 0.25% trypsin in 0.01% EDTA whenever they became confluent. All assays in the present study were done at temperatures of 37°C, 95% sterile air and 5% CO_2_ in a saturation humidified incubator. Cells were treated with leptin (100 ng/mL, BioVendor, Germany) for 24 h in the presence or absence of the mineralocorticoid receptor antagonist (eplerenone 10^-6^ mol/L; Sigma; St Louis, M O, USA), and in the presence or absence of the inhibitors of either PI3K or MEK pathwaysLY294002 (20 x10^-6^ mol/L) and PD98059 (25 x10^-6^ mol/L), respectively.

*Measurement of activation of PI3/Akt and ERK signaling mediators*

Cells were treated with leptin (100 ng/mL, BioVendor, Germany) for different periods of time (0-60 minutes).

*Western blot*

Total proteins were prepared as previously described^2^ from cell extracts isolated from cardiac myofibroblasts. Proteins were separated by SDS-PAGE on 12.5% polyacrylamide gels and transferred to polyvinylidene difluoride membranes (Hybond-P; Amersham Biosciences, Piscataway, NJ). Membranes were probed with primary antibody for collagen I (AbD Serotec, Oxford, UK; dilution: 1:1000), connective tissue growth factor (CTGF; Torrey Pines Biolabs Inc., East Orange, NJ; dilution: 1:1000), transforming growth factor β (TGF-β; Abcam plc, Cambridge, UK; dilution: 1:1000), galectin-3 (Epitomics, Burlingame, CA, USA; dilution 1:1000), phosphorylated (Ser473) and total Akt (Cell signaling; Boston, MA, USA; dilution: 1:500), phosphorylated (Cell Signaling Technology, Inc, New England, USA: 1:500) and total (Zymed Laboratories, CA, USA; dilution 1:500) Erk1/2, phosphorylated (Ser 727.25; dilution 1:500) and total STAT3 ( Santa Cruz Biotechnology, Inc, Dallas, TX; dilution 1:500)and β-actin (Sigma; dilution: 1:10,000) as a loading control. Signals were detected using the ECL system (Amersham Pharmacia Biotech; Piscataway, NJ, USA). Results are expressed as an n-fold increase over the values of the control group in densitometric arbitrary units.

*Measurement of intracellular superoxide anion production*

The oxidative fluorescent dye dihydroethidium (DHE; Invitrogen) was used to evaluate the production of ion superoxide (O_2_^.-^). Cells were incubated for 24 hours with either vehicle or leptin (100 ng/mL, BioVendor, Germany) in the presence or absence of the mineralocorticoid receptor antagonist (eplerenone 10^-6^ mol/L; Sigma; St Louis, MO, USA), and in the presence or absence of the inhibitors of either PI3K or MEK pathways (LY294002 and PD98059, respectively). Cells were then incubated with 5 × 10^−6^ mol/L DHE for 30 min in a light-protected humidified chamber at 37 °C. Cells were subsequently washed with warm phosphate-buffered saline (PBS) and analyzed with a 40× objective in a fluorescent laser scanning Leica DMI 3000 microscope and quantified by using an image analyzer (LAS, LEICA).

For each condition 150–200 cells were analyzed with a 40X objective (Leica DM 2000; Leica Camera AG, Wetzlar, Germany) and quantified by using an image analyzer (LAS, LEICA. Results are expressed as an n-fold increase over the values of the control group. A single researcher unaware of the experimental groups performed the analysis. Three different assays were each performed in quintuplicate

*Measurement of mitochondrial superoxide anion production, intracellular reactive nitrite species and mitochondrial inner transmembrane potential detection*

For detection of mitochondrial O_2_^.-^ production, cardiac myofibroblasts were stimulated at 37ºC with 100 ng/mL of leptin at the indicated times, or with different doses of leptin for 24 h. After that, cells were washed and loaded with 4 x10^-6^ mol/L of MitoSOX^TM^ Red for 10 minutes, at 37ºC. For detection of intracellular nitric oxide (NO) production, cardiac myofibroblasts were preloaded with 5 x10^-6^mol/L of 4-Amino-5-methylamino-2′,7′-difluorofluorescein (DAF-FM) diacetate for 30 min at 37ºC, washed, and then stimulated with leptin at the indicated times and doses. Fluorescent signals derived by reaction of MitoSOX^TM^ Red or DAF-FM with ROS/RNS were analyzed by recording FL2 and FL1 fluorescence, respectively, in a Gallios^TM^ flow cytometer (Beckman Coulter). In some experiments, cells were pretreated for 30 min with the mineralocorticoid receptor antagonist (eplerenone 10^-6^ mol/L; Sigma; St Louis, MO, USA) before incubation with leptin (100 ng/mL).

To evaluate mitochondrial transmembrane potential (ΔΨm), cardiac myofibroblasts were stimulated with 100 ng/mL of leptin or 500 x10^-6^ mol/L of H_2_O_2_ for 24 h at 37ºC. After treatment, cells were incubated with 4 μmol/L rhodamine 123 or with 5x10^-6^ μmol/L of 5, 5′, 6, 6′-tetrachloro-1, 1′,3,3′-tetraethylbenzimidazolo carbocyanine iodide (JC-1) for 30 min at room temperature. Loaded cells were washed with PBS and changes in fluorescence were monitored using flow cytometry analysis.

Experiments were repeated at least three times. The data are given as one representative histogram. Data analysis was performed using WinMDI 2.7 software.

*EGFR transactivation*

Cardiac myofibroblasts, 5x10^6^/flask, were treated with 100 ng/mL of leptin for the indicated times at 37 ºC. Then, cells were fixed in 4% paraformaldehyde for 15 min, washed with PBS and permeabilized with 0.3% Triton X-100 for 5 min. After that, cells were incubated with anti-phospho EGFR (Tyr1173) or EGFR (Tyr845) antibody (Santa Cruz Biotechnology Inc, Santa Cruz, CA, USA; dilution 1:50) for 1 h at 4°C, and then with a FITC-labelled secondary antibody for 45 min at 4°C. After washing, the cells were analyzed with a Flow Cytometer (Gallios^TM^; Beckman Coulter, USA). Data analysis was performed using WinMDI 2.7 software.

*Cell proliferation assay*

Cell proliferation was assessed using the Cell Titer 96 Non-Radioactive Cell Proliferation Assay (Promega Corporation, Madison, WI, USA). Cardiac myofibroblasts were seeded on 96-well plates (20 × 10^3^ cells/well) in DMEM medium and were allowed to attach for 24-36 hours. Afterwards, cells were switched to serum-free medium for 24-h. Cells were then treated with leptin (10-100 ng/ml), aldosterone (10^-9^-10^-6^ mol/L) and a combination of them or vehicle for 24-48 hours. The proliferative response was quantified by adding MTS tetrazolium solution (20μl/well). After 2- 3 hours of incubation absorbance was measured at 490 nm in a microplate reader (ASYS Hitech GmbH, Austria). Three different assays were each performed in quintuplicate.

*Animals*

Male Wistar rats of 150g (Harlan Ibérica, Barcelona, Spain) were fed either an high-fat diet (HFD, 35% fat; Harlan Teklad #TD.03307, Haslett, MI, USA; n=16) or a standard diet (3.5% fat; Harlan Teklad #TD.2014; Haslett, MI, USA; =16) for 6 weeks. Half of the animals of each group received the mitochondrial antioxidant MitoTempo (0.7 mg Kg^−1^ day ^−1^ Sigma, i.p.; Louis, MO, USA) or vehicle (saline) from the third week on. The dose used of MitoTempo was chosen from previous publications.^3^ Animal weight was controlled every week. Food and water intake were administered *ad libitum* and determined throughout the experimental period. Blood and heart were collected at the end of the experiment. The Animal Care and Use Committee of Universidad Complutense de Madrid and Dirección General de Medio Ambiente, Comunidad de Madrid (PROEX 242/15) approved all experimental procedures according to the Spanish Policy for Animal Protection RD53/2013, which meets the European Union Directive 2010/63/UE.

### ***Evaluation of cardiac function***

Cardiac function was evaluated by transthoracic echocardiography with an Acuson Sequoia 256 (Siemens Medical Solutions, Germany) connected to a 15-MHz linear transducer. 2D-guided M-mode recordings were made from short axis views to measure left ventricular (LV) chamber dimensions, interventricular septum and posterior wall thickness. LV diastolic (LVDA) and systolic (LVSA) areas were measured from the bidimentional parasternal long-axis view. The mean measurements from several consecutive beats were used for data analysis. LV chamber volumes were calculated using the cylindrical model. This model assumes the ventricle is approximated by a cylinder. Left ventricular ejection fraction (EF) was calculated according to the Teicholz Formula: ([EDD](http://www.echobyweb.com/htm_level3_outofstructure/formulas&calculations/lv_systolic_function_mmode_eng_01.htm)^3^ x 7)/(2.4 + [EDD](http://www.echobyweb.com/htm_level3_outofstructure/formulas&calculations/lv_systolic_function_mmode_eng_01.htm)) and LV systolic chamber function Diastolic function was assessed by the E/A ratio, the E-wave (pump function) was determined from LV endocardial fractional shortening (FS) =(LVEDD-LVESD)/LVEDD x 100.

The diastolic function was assessed by early and late transmitral peak diastolic flow velocity (E and A waves) and ratio between E-waves and A-wave (E/A) was calculated.

# *Histological Analysis*

Cardiac tissue samples were dehydrated, embedded in paraffin and cut in 4 μm-thick sections. Fibrosis was quantified in picrosirius red-stained sections using an analysis system (LAS, LEICA). The area of interstitial fibrosis was identified after excluding the vessel area from the region of interest, as the ratio of interstitial fibrosis or collagen deposition to the total tissue area. For each sample, 10 to 15 fields were analyzed with a 40X objective under transmitted light microscope. A single researcher unaware of the experimental groups performed the analysis.

*Detection of cardiac superoxide anion production*

The oxidative fluorescent dye dihydroethidium (DHE) was used to evaluate production of O_2_^.-^. Briefly, cardiac sections (14 μm) were equilibrated in Krebs-HEPES buffer (in mmol/L: NaCl 130, KCl 5.6, CaCl_2_ 2, MgCl_2_ 0.24, HEPES 8.3, glucose 11, pH 7.4). Fresh buffer containing DHE (5x10^-6^ mol/L, 30 min, 37 ºC) was then added and viewed by fluorescent laser scanning microscope (40X objective in a Leica DMI 3000 microscope) (Ex561 nm and Em610 nm) using the same imaging settings in each case.

The mean fluorescence densities of 10 to 15 fields per animal were analyzed with a 40X objective (Leica DM 2000; Leica Camera AG, Wetzlar, Germany) and quantified by using an image analyzer (LAS, LEICA). A single researcher that was unaware of the experimental groups performed the analysis.

*Measurement of circulating leptin and aldosterone levels*

Serum leptin and aldosterone levels were measured using a specific quantitative sandwich enzyme immunoassay according to the manufacturer's instructions (Biovendor, Germany Chemical Company and Cayman Chemical Company; Ann Arbor; Mi, USA, respectively).

*Statistical analysis*

Data are expressed as mean ± SEM. Normality of distributions was verified by means of the Kolmogorov–Smirnov test. Data were analyzed using a one-way analysis of variance, followed by a Newman–Keuls to assess specific differences among groups or conditions or unpaired Student’s t-test as corresponding using Graph Pad Software Inc. (San Diego, CA, USA). The predetermined significance level was p < 0.05.

**Figures**

**Figure S1. Effect of mineralocorticoid receptor antagonist on reactive-nitrite species (RNS) production induced by leptin in cardiac myofibroblasts.** Cardiac myofibroblasts stained with DAF-FM diacetate and stimulated with leptin (100 ng/mL). (a) Time course of RNS generation. Representative histogram. (b) Flow cytometry analysis of RNS: Mean fluorescence intensity (MFI). (c) RNS production in presence or absence of the mineralocorticoid receptor antagonist (eplerenone; 10^-6^ mol/L) after 24 hours of treatment. Representative histogram. Untreated cells (solid black curves) were compared with cells treated with leptin (open dark blue curves) or with eplerenone + leptin (light-blue curves). Bar graphs represent the mean ± SEM of 3 assays. * p<0.05 *vs.* 0 time.

**Figure S2. Effect of leptin on mitochondrial membrane potential in cardiac myofibroblasts.** Cardiac myofibroblasts stained with either JC-1 (a) or Rhodamine 123 (b) and treated with leptin (100 ng/mL) or H_2_O_2_ (500 x10^-6^ mol/L) for 24 hours. Untreated cells (solid curves) were compared with stimulated cells (open curves). Representative of 3 experiments

**Figure S3. Effect of leptin on Akt and MEK pathways in cardiac myofibroblasts.** Protein levels of (a) pAkt/Akt and (b) pERK 1/2/ERK1/2 in cardiac myofibroblasts stimulated by leptin (100 ng/mL) for 24 hours. Bar graphs represent the mean ± SD of 3-4 assays in arbitrary. *p<0.05; ** p<0.001 *vs.* control. Uncropped images of the blots for Figures S3 [and](https://www.nature.com/articles/s41598-017-04384-3#Fig1) 3B are shown in supplementary Figure 6.

**Figure S4. Effect of leptin on STAT3 phosphorylation and impact of inhibition of Akt and MEK pathways in cardiac myofibroblasts.** Protein levels of pSTAT3/STAT3 in cardiac myofibroblasts stimulated by leptin (100 ng/mL) (a) for indicated time intervals or (b) in the presence or absence of the inhibitors of either MEK (PD98059; PD; 25x10^-6^ mol/L) or Akt (LY294002; LY; 20x10^-6^ mol/L) pathways for 24 hours. Bar graphs represent the mean ± SD of 3-4 assays in arbitrary units. **p<0.01; *** p<0.001 *vs.* control. ††p<0.01*vs.* leptin

**Lep 100 ng/mL - + - +**

**Aldo 10^-6^mol/L - - + +**

**Figure S5. Effect of leptin and aldosterone on the proliferative activity of cardiac myofibroblasts.** Cardiac myofibroblasts were stimulated with leptin (100 ng/mL; Lep) in the absence or the presence of aldosterone (10^-6^ mol/L; Aldo) for 24 hours. Proliferation was determined by an MTT assay. Data are expressed as percent of unstimulated cells. Values are mean±SEM of three assays.

**Figure S6. Original immunoblots for indicated figures.**

**References**

1 Martin, R. *et al.* DIOL triterpenes block profibrotic effects of angiotensin II and protect from cardiac hypertrophy. *PLoS One* **7**, e41545, doi:10.1371/journal.pone.0041545 (2012).

2 Martinez-Martinez, E. *et al.* The lysyl oxidase inhibitor (beta-aminopropionitrile) reduces leptin profibrotic effects and ameliorates cardiovascular remodeling in diet-induced obesity in rats. *J Mol Cell Cardiol* **92**, 96-104, doi:10.1016/j.yjmcc.2016.01.012 (2016).

3 Garcia-Redondo, A. B. *et al.* c-Src, ERK1/2 and Rho kinase mediate hydrogen peroxide-induced vascular contraction in hypertension: role of TXA2, NAD(P)H oxidase and mitochondria. *J Hypertens* **33**, 77-87, doi:10.1097/HJH.0000000000000383 (2015).
